# Transcriptome profiling of the small intestinal epithelium in germfree versus conventional piglets

**DOI:** 10.1186/1471-2164-8-215

**Published:** 2007-07-05

**Authors:** Shankar R Chowdhury, Dale E King, Benjamin P Willing, Mark R Band, Jonathan E Beever, Adrienne B Lane, Juan J Loor, Juan C Marini, Laurie A Rund, Lawrence B Schook, Andrew G Van Kessel, H Rex Gaskins

**Affiliations:** 1 Department of Animal Sciences, University of Illinois, Urbana, Illinois, 61801, USA; 2 Department of Animal and Poultry Science, University of Saskatchewan, Saskatoon, S7N 5A8, Canada; 3 W. M. Keck Center for Comparative and Functional Genomics, University of Illinois, Urbana, Illinois, 61801, USA; 4 Division of Nutritional Sciences, University of Illinois, Urbana, Illinois, 61801, USA; 5 U.S. Department of Agriculture/ARS Children's Nutrition Research Center, Department of Pediatrics, Baylor College of Medicine, Houston, Texas, 77030, USA; 6 Department of Pathobiology, University of Illinois, Urbana, Illinois, 61801, USA; 7 Institute for Genomic Biology, University of Illinois, Urbana, Illinois, 61801, USA

## Abstract

**Background:**

To gain insight into host-microbe interactions in a piglet model, a functional genomics approach was used to address the working hypothesis that transcriptionally regulated genes associated with promoting epithelial barrier function are activated as a defensive response to the intestinal microbiota. Cesarean-derived germfree (GF) newborn piglets were colonized with adult swine feces, and villus and crypt epithelial cell transcriptomes from colonized and GF neonatal piglets were compared using laser-capture microdissection and high-density porcine oligonucleotide microarray technology.

**Results:**

Consistent with our hypothesis, resident microbiota induced the expression of genes contributing to intestinal epithelial cell turnover, mucus biosynthesis, and priming of the immune system. Furthermore, differential expression of genes associated with antigen presentation (pan SLA class I, *B2M*, *TAP1 *and *TAPBP*) demonstrated that microbiota induced immune responses using a distinct regulatory mechanism common for these genes. Specifically, gene network analysis revealed that microbial colonization activated both type I (IFNAR) and type II (IFNGR) interferon receptor mediated signaling cascades leading to enhanced expression of signal transducer and activator of transcription 1 (STAT1), STAT2 and IFN regulatory factor 7 (IRF7) transcription factors and the induction of IFN-inducible genes as a reflection of intestinal epithelial inflammation. In addition, activated RNA expression of NF-kappa-B inhibitor alpha (*NFκBIA*; a.k.a I-kappa-B-alpha, IKBα) and toll interacting protein (*TOLLIP*), both inhibitors of inflammation, along with downregulated expression of the immunoregulatory transcription factor GATA binding protein-1 (*GATA1*) is consistent with the maintenance of intestinal homeostasis.

**Conclusion:**

This study supports the concept that the intestinal epithelium has evolved to maintain a physiological state of inflammation with respect to continuous microbial exposure, which serves to sustain a tight intestinal barrier while preventing overt inflammatory responses that would compromise barrier function.

## Background

The gastrointestinal (GI) tract of the pig harbors a numerically dense and metabolically active microbiota comprised mainly of bacteria [1]. Indeed, all animals have, and seemingly require, long-term cooperative associations with indigenous bacteria in the GI tract. Studies with gnotobiotic animal models demonstrate most conclusively that indigenous bacteria stimulate the normal maturation of host tissues and provide key defense and nutritional functions [2]. This mutualistic relationship has been selected over evolutionary time resulting in a stable microbiota in mature animals that is generally similar in composition and function in a diverse range of animal species [3].

Despite evolutionary stability, the intestinal microbiota develops in individual animals in a characteristic successional pattern that requires substantial adaptation by the host during early life. The impact of the developing microbiota as well as the metabolic activities of climax communities require special consideration when viewed in the context of pig production in which efficiency of animal growth is a primary objective [4].

The epithelial lining of the GI tract is characterized by a high cell turnover rate and the constant production of a protective mucus coat. Together these two physiological processes provide effective innate defense against luminal threats including those emanating from normal gut bacteria. In fact, epithelial cell turnover and secretory activity are both profoundly affected by the numbers, types, and spatial distribution of GI bacteria, with the latter microbial features being influenced by both exogenous and endogenous (host-derived) nutrients. Innate defense functions afforded by the epithelium are provided at the expense of animal growth efficiency. Specifically, GI tissues represent only 5% of body weight (approximate) but they receive a disproportionate fraction of cardiac output and contribute 15–35% of whole body oxygen consumption and protein turnover [5-7]. Only 10% of the total protein synthesized by the GI tract is accumulated as new mass [8]; most proteins are lost in sloughed epithelial cells or as secreted products such as mucus.

Carriage of microbial populations capable of utilizing refractory plant components enabled feral pigs to exploit distinct habitats thereby enhancing survival and reproductive success. Animal growth efficiency is, however, a concept introduced only upon domestication of the pig as a food animal. These issues provoke consideration of an optimal gut microbiota for intestinal health versus its effects on the efficiency of gastrointestinal and whole body growth throughout the productive life cycle of a pig. However, the normal microbiota of the pig intestine has received surprisingly little attention from an animal growth perspective. In the present study, laser capture microdissection (LCM) and microarray analysis were used to distinguish gene expression profiles in the small intestinal epithelium of GF versus conventional (CONV) neonatal piglets. The genetic pathways induced are consistent with the concept that the host responds to normal gut bacteria by mounting epithelial defenses that presumably impact the efficiency of whole animal growth.

## Results

### Microbial status and animal performance

Bacterial colony growth was not observed on cultures of fecal swabs and cecal digesta of GF pigs indicating GF status was maintained. Cecal colonization (log CFU/g contents) in CONV pigs ranged from 8.1 to 9.5 and 8.5 to 9.7 for total aerobes and anaerobes, respectively. All pigs appeared healthy with body weight gain (kg ± SE) over the 14 day period of 1.26 ± 0.23 and 1.36 ± 0.17 for GF and CONV pigs, respectively.

### Principal component analysis

PCA analysis demonstrated that 60% of the variation in gene expression [component 1 (32%) and component 2 (28%); see Additional file 1] was due to the state of microbial colonization (CONV versus GF). In contrast, 25% of the variation in gene expression (component 3) was due to an epithelial compartment effect (crypt versus villi). Fifteen percent of the variation (component 4) did not follow a distinct pattern of treatment or compartment effect, perhaps indicating a treatment-compartment interaction, variation due to the technical procedures involved in sample preparation, sample variation (among piglets) or a combination of these factors [see Additional file 1]. Thus, most of the variation in gene expression was due to the treatment effect with minor effects contributed by the epithelial-compartment and treatment-compartment interactions.

### Gene expression in crypt versus villi

Seventy genes, involved in transcription, cell proliferation and differentiation, nutrient transport and metabolism, and immune responses were differentially expressed (*P *< 0.05) in CONV crypts compared with GF crypts [see Additional file 2]. Twelve genes, mainly involved in immune responsiveness, were significantly upregulated in CONV versus GF villi [see Additional file 3]. These results indicate that, relative to proliferative crypt epithelial cells, differentiated and short-lived villus epithelial cells are less responsive to microbial colonization-induced gene expression. Because the state of microbial colonization elicited the greatest responses from the intestinal epithelium, subsequent analyses focused on the effects of intestinal microbiota irrespective of crypt versus villus compartment effects.

### Microbiota effects on intestinal epithelial gene expression

A total of 223 genes were differentially expressed (*P *< 0.05) in the intestinal epithelium (crypt plus villi) of CONV relative to GF animals. Among them, 170 genes were upregulated, and 53 genes were downregulated in CONV compared to GF epithelia. Differentially expressed genes were categorized according to gene ontology (GO) biological processes: 10% in transcription, 9% in signal transduction, 6% in cell proliferation, differentiation and regulation of cell growth, 5% in transport, 10% in metabolism, 4% in electron transport mechanisms, 5% in immune responsiveness, 23% were involved in 'other' processes and 28% encode genes of unknown function [see Additional file 4], which is consistent with known limitations in gene annotation in the current GO database of porcine genes [9].

### Gene transcription

CONV animals exhibited increased expression of several genes encoding proteins involved in transcriptional processes such as DNA directed RNA polymerase-II (*POLR2J2*), *Drosophila *sine oculis homeobox homolog 6 (*SIX6*), signal transducer and activator of transcription-1 (*STAT1*), transcriptional adaptor 3-like (*TADA3L*), and zinc finger protein-398 (*ZNF398*) (Table 1). Furthermore, microbial colonization induced mRNA expression of retinoid × receptor beta (*RXRB*), HLA-B associated transcript-4 (*BAT4*), and pre-B-cell leukemia transcription factor-2 (*PBX2*), which are each encoded within the major histocompatibility complex (MHC) and may therefore be involved in some aspect of immunity [10]. On the other hand, CONV animals exhibited lower expression of genes encoding GATA binding protein-1 (*GATA1*), transcription elongation factor A (SII)-like-8 (*TCEAL8*) and methyl-CpG-binding protein-2 (*MECP2*, a negative regulator of transcription) (Table 1).

**Table 1:** Intestinal epithelial gene expression modulated by microbiota in CONV versus GF piglets

Functional class	Unigene ID	Gene description	FDR adjusted *P *value	Fold change^1^
Transcription	Hs.444468	CTD (carboxy-terminal domain) small phosphatase 1 (*CTDSP1*)	0.007	1.57
	Hs.433879	DNA directed RNA polymerase II polypeptide J-related gene (*POLR2J2*)	0.001	1.33
	Hs.509226	FK506 binding protein 3, 25 kDa (*FKBP3*)	0.029	0.67
	Hs.765	GATA binding protein 1 (globin transcription factor 1, *GATA1*)	0.006	0.70
	Hs.247478	HLA-B associated transcript 4 (*BAT4*)	0.042	1.24
	Hs.535030	Hypothetical protein MGC13098 (*MGC13098*)	0.046	1.64
	Hs.200716	Methyl CpG binding protein 2 (*MECP2*)	0.007	0.65
	Hs.509545	Pre-B-cell leukemia transcription factor 2 (*PBX2*)	0.007	1.51
	Hs.643558	Retinoblastoma binding protein 4 (*RBBP4*)	0.003	1.47
	Hs.388034	Retinoid × receptor, beta (*RXRB*)	0.003	1.41
	Hs.470412	RNA binding motif, single stranded interacting protein 1 (*RBMS1*)	0.018	1.39
	Hs.433343	Serine/arginine repetitive matrix 2 (*SRRM2*)	0.001	1.46
	Hs.470943	Signal transducer and activator of transcription 1 (*STAT1*)	0.001	1.86
	Hs.194756	Sine oculis homeobox homolog 6 (*SIX6*)	0.008	1.86
	Hs.525091	Testis-specific kinase 2 (*TESK2*)	0.006	1.24
	Hs.369762	Thymidylate synthetase (*TYMS*)	0.043	0.71
	Hs.389734	Transcription elongation factor A (SII)-like 8 (*TCEAL8*)	0.007	0.62
	Hs.386390	Transcriptional adaptor 3 (NGG1 homolog, yeast)-like (*TADA3L*)	0.007	1.58
	Hs.517296	V-ets erythroblastosis virus E26 oncogene homolog 2 (*ETS2*)	0.002	1.40
	Hs.490504	Zinc finger protein 398 (*ZNF398*)	0.017	1.39
	Hs.438536	Zinc finger protein 705A (*ZNF705A*)	0.017	1.42
	Hs.371794	Zinc finger, NFX1-type containing 1 (*ZNFX1*)	0.007	1.67

Signal transduction	Hs.371240	A kinase (PRKA) anchor protein 12 (*AKAP7*)	0.003	1.77
	Hs.150423	Cyclin-dependent kinase 9 (CDC2-related kinase) (*CDK9*)	0.039	1.59
	Hs.173135	Dual-specificity tyrosine phosphorylation regulated kinase 2 (*DYRK2*)	0.042	0.62
	Hs.515415	Inositol 1,4,5-trisphosphate 3-kinase C (*ITPKC*)	0.033	1.37
	Hs.529400	Interferon (alpha, beta and omega) receptor 1 (*IFNAR1*)	0.013	1.53
	Hs.520414	Interferon (gamma) receptor 1 (*IFNGR1*)	0.030	1.52
	Hs.433442	Kinesin-associated protein 3 (*KIFAP3*)	0.012	1.53
	Hs.133421	Leukemia inhibitory factor receptor (*LIFR*)	0.003	2.31
	Hs.485262	Mitochondrial carrier homolog 1 (*MTCH1*)	0.034	1.62
	Hs.419640	Parkinson disease 7 (*PARK7*)	0.030	0.75
	Hs.509067	Platelet-derived growth factor receptor, beta polypeptide (*PDGFRB*)	0.038	1.24
	Hs.2890	Protein kinase C, gamma (*PRKCG*)	0.035	0.59
	Hs.513683	Protein serine kinase H1 (*PSKH1*)	0.041	1.35
	Hs.458417	Regulator of G-protein signalling 8 (*RGS8*)	0.036	1.45
	Hs.88012	signaling threshold regulating transmembrane adaptor 1 (*SIT1*)	0.038	1.47
	Hs.470943	Signal transducer and activator of transcription 1 (*STAT1*)	0.001	1.86
	Hs.441498	Signal transducing adaptor molecule 1 (*STAM1*)	0.014	1.29
	Hs.525091	Testis-specific kinase 2 (*TESK2*)	0.006	1.25
	Hs.355899	Tumor necrosis factor receptor superfamily, member 12A (*TNFRSF12A*)	0.021	0.78
	Hs.47061	Unc-51-like kinase 1 (*ULK1*)	0.040	1.52
	Hs.646283	Virus-induced signaling adapter (*VISA*)	0.001	1.43
	Hs.512079	WAS protein family, member 2 (*WASF2*)	0.003	2.06

Cell proliferation, differentiation	Hs.515371	Calpain, small subunit 1 (*CAPNS1*)	0.033	0.68
	Hs.443625	Collagen, type III, alpha 1 (*COL3A1*)	0.012	1.14
	Hs.150423	Cyclin-dependent kinase 9 (CDC2-related kinase) (*CDK9*)	0.039	1.59
	Hs.437379	Development and differentiation enhancing factor-like 1 (*DDEFL1*)	0.013	1.30
	Hs.130316	Drebrin 1 (*DBN1*)	0.045	1.53
	Hs.26770	Fatty acid binding protein 7 (*FABP7*)	0.011	0.64
	Hs.87752	Moesin (*MSN*)	0.031	1.35
	Hs.516633	NCK-associated protein 1 (*NCKAP1*)	0.013	0.75
	Hs.191346	Septin 7 (*SEPT7*)	0.024	0.75
	Hs.530477	Signal-induced proliferation-associated gene 1 (*SIPA1*)	0.001	1.56
	Hs.527973	Suppressor of cytokine signaling 3 (*SOCS3*)	0.045	1.30
	Hs.386390	Transcriptional adaptor 3 like (*TADA3L*)	0.015	1.58
	Hs.355899	Tumor necrosis factor receptor superfamily, member 12A (*TNFRSF12A*)	0.021	0.78

Transport	Hs.518060	ADP-ribosylation-like factor 6 interacting protein 5 (*ARL6IP5*)	0.009	0.69
	Hs.511311	ATPase, Class I, type 8B, member 4 (*ATP8B4*)	0.037	2.32
	Hs.86905	ATPase, lysosomal, V1 subunit C, isoform 1 (*ATP6V1C1*)	0.043	2.10
	Hs.503721	Dynein, cytoplasmic, heavy polypeptide 2 (*DNCH2*)	0.021	0.53
	Hs.26770	Fatty acid binding protein 7 (*FABP7*)	0.011	0.64
	Hs.388668	Kelch-like 2 (*KLHL2*)	0.026	0.50
	Hs.645375	Mitochondrial carrier family protein (*SLC25A*)	0.015	0.70
	Hs.477361	SEC22 vesicle trafficking protein homolog A (*SEC22A*)	0.041	0.61
	Hs.458917	Secretory carrier membrane protein 2 (*SCAMP2*)	0.042	1.44
	Hs.14846	Solute carrier family 7 (*SLC7A1*)	0.026	1.24
	Hs.462379	Target of myb1-like 2 (*TOM1L2*)	0.004	1.59
	Hs.352018	Transporter 1, ATP-binding cassette, sub-family B (*TAP1*)	0.001	3.08

Metabolism	Hs.591852	ADAM metallopeptidase domain 9 (*ADAM9*)	0.021	1.88
	Hs.465720	Acyl-CoA synthetase bubblegum family member 2 (*ACSBG2*)	0.021	1.31
	Hs.81934	Acyl-Coenzyme A dehydrogenase, short/branched chain (*ACADSB*)	0.012	1.98
	Hs.88778	Carbonyl reductase 1 (*CBR1*)	0.037	1.34
	Hs.437379	Development and differentiation enhancing factor-like 1 (*DDEFL1*)	0.013	1.30
	Hs.463089	Dodecenoyl-Coenzyme A delta isomerase (*DCI*)	0.038	1.37
	Hs.369762	Enolase superfamily member 1 (*ENOSF1*)	0.043	0.71
	Hs.79322	Glutaminyl-tRNA synthetase (*QARS*)	0.035	1.37
	Hs.647690	Glyceraldehyde 3-phosphate dehydrogenase (*GAPDH*)	0.003	1.27
	Hs.524418	Glycerol-3-phosphate dehydrogenase 1 (*GPD1*)	0.010	0.44
	Hs.180878	Lipoprotein lipase (*LPL*)	0.002	0.65
	Hs.162757	Low density lipoprotein-related protein 1 (*LRP1*)	0.036	0.68
	Hs.514713	Metallophosphoesterase 1 (*MPPE1*)	0.047	1.39
	Hs.94949	Methylmalonyl CoA epimerase (*MCEE*)	0.039	0.54
	Hs.518424	NADH dehydrogenase 1 beta subcomplex, 5 (*NDUFB5*)	0.008	0.49
	Hs.12851	Phosphatidylserine synthase 2 (*PTDSS2*)	0.036	1.34
	Hs.368157	Phosphorylase, glycogen (*PYGB*)	0.037	1.34
	Hs.98381	Protease, serine, 35 (*PRSS35*)	0.010	0.67
	Hs.471441	Proteasome subunit, beta type, 2 (*PSMB2*)	0.043	0.71
	Hs.548558	Similar to cathepsin D (*CTSD*)	0.034	1.55
	Hs.156668	Ubiquinone oxidoreductase complex B15 subunit (*NDUFB4*)	0.002	2.21
	Hs.272011	UDP-Gal:betaGlcNAc beta 1,4-galactosyltransferase, polypeptide 1 (*B4GALT1*)	0.037	1.26

Electron transport	Hs.81934	Acyl-Coenzyme A dehydrogenase, short/branched chain (*ACADSB*)	0.012	1.98
	Hs.201667	Aldo-keto reductase family 1, member D1 (*AKR1D1*)	0.015	2.51
	Hs.433901	Cytochrome c oxidase subunit 8A (ubiquitous) (*COX8A*)	0.021	1.42
	Hs.511367	Cytochrome P450, family 19, subfamily A, polypeptide 1 (*CYP19A1*)	0.029	1.79
	Hs.642706	Flavin containing monooxygenase 5 (*FMO5*)	0.013	1.72
	Hs.211046	Hypothetical protein LOC126248 (*LOC126248*)	0.002	2.24
	Hs.518424	NADH dehydrogenase 1 beta subcomplex, 5 (*NDUFB5*)	0.008	0.49
	Hs.125221	Thioredoxin domain containing 1 (*TXNDC1*)	0.003	1.48

Immune response	Hs.529019	Bactericidal/permeability-increasing protein (*BPI*)	0.002	2.02
	Hs.534255	Beta-2-microglobulin (*B2M*)	0.021	1.92
	Hs.504641	CD163 antigen (*CD163*)	0.023	1.29
	Hs.278694	CD209 antigen (*CD209*)	0.003	1.47
	Hs.372679	Fc fragment of IgG, low affinity IIIb, receptor (CD16b) (*FCGR3B*)	0.050	1.66
	Hs.3268	Heat shock 70 kDa protein (HSP70B, *HSPA6*)	0.001	1.28
	Hs.497723	Hypothetical protein MGC27165 (*MGC27165*)	0.011	1.42
	Hs.449585	Immunoglobulin lambda locus (*IGL@*)	0.001	3.03
	Hs.389724	Interferon-induced protein 44-like (*IFI44L*)	0.001	2.53
	Hs.77961	Major histocompatibility complex, class I, B (*HLA-B*)	0.001	2.44
	Hs.548432	Similar to CD63 antigen (*CD63*)	0.016	0.49
	Hs.370937	TAP binding protein (tapasin, *TAPBP*)	0.031	1.91
	Hs.352018	Transporter 1, ATP-binding cassette, sub-family B (*TAP1*)	0.001	3.08

^1^Fold change is the ratio of CONV versus GF.

### Signal transduction

Most of the differentially expressed genes involved in signal transduction processes were upregulated in CONV animals [e.g., *STAT1*, leukemia inhibitory factor receptor alpha (*LIFR*), signaling threshold regulating transmembrane adaptor 1 (*SIT1*), interferon (IFN) αβ receptor-1 (*IFNAR1*), IFNγ receptor-1 (*IFNGR1*), platelet-derived growth factor receptor B(*PDGFRB*), kinesin-associated protein-3 (*KIFAP3*), and regulator of G-protein signaling-8 (*RGS8*)] (Table 1), indicating that receptor-mediated signaling cascades were activated by mutualistic microbes. The gene encoding protein kinase C gamma (PKC-γ) was downregulated (*P *< 0.05) in CONV versus GF epithelia. Intestinal epithelial cells express twelve known isoforms of PKC that play critical roles in intracellular signaling including cell proliferation, differentiation, apoptosis, adhesion, membrane remodeling, migration, ion secretion, and barrier function [11]. However, PKC-γ is one of the least explored isoforms of the PKC family and further studies are necessary to determine its involvement in the transduction of signals activated by intestinal microbes in ileal epithelial cells.

### Cell proliferation and differentiation

Bacterial colonization downregulated the expression of various genes encoding proteins involved in cell apoptosis [NCK-associated protein-1 (*NCKAP1*), TNF receptor superfamily, member 12A (*TNFRSF12A*)], downregulation of cell proliferation (fatty acid bind protein-7; *FABP7*) and cytokinesis (septin 7; *SEPT7*) (Table 1). In addition, genes involved in cell proliferation [cyclin-dependent kinase-9 (*CDK9*), signal-induced proliferation associated gene-1 (*SIPA1*), *PDGFRB *[12], suppressor of cytokine signaling-3 (*SOCS3*)], differentiation (drebrin1; *DBN1*) and regulation of cell growth (development and differentiation enhancing factor-like-1; *DDEFL1*) and structural integrity (moesin; *MSN*) were upregulated in CONV compared with GF piglets (*P *< 0.05) indicating the involvement of a biotic stimulus that induces genes associated with epithelial cell turnover to protect the host from microbial colonization.

### Transport and metabolism

Genes encoding ATPase and solute carrier family-7 proteins, which are associated with cationic amino acid and monocarboxylic acid transport, were upregulated by intestinal microbiota (Table 1). Expression of genes encoding Kelch-like-2 protein (intracellular protein transporter) and fatty acid binding protein (transport of long chain fatty acids), was significantly downregulated in CONV piglets indicating differential expression of transport systems in CONV versus GF pigs. Intestinal microbiota downregulated glycerol-3-phosphate dehydrogenase 1 (*GPD1*) expression whose product is involved in fructose metabolism. Genes encoding glycogen phosphorylase and glyceraldehyde-3-phosphate dehydrogenase associated with glycogenolysis, gluconeogenesis, and glycolysis were significantly upregulated in CONV compared to GF animals, perhaps demonstrating a higher energy requirement of CONV epithelial cells. With respect to lipid metabolism, expression of genes encoding acyl-coenzyme-A-synthetase and acyl-coenzyme-A-dehydrogenase related to fatty acid metabolism was significantly upregulated by the microbiota presumably to increase cellular energy supply. Nutrient requirements of GF rodents are low compared to those colonized with a normal microbiota [13].

Resident microbiota inhibited expression of serine protease-35 (*PRSS35*) (*P *< 0.05). The RNA expression of the gene encoding the lysosomal protease cathepsin D (*CTSD*) was significantly upregulated in CONV piglets. Furthermore, two genes encoding phosphatidylserine synthase-2 and glutamyl-tRNA synthetase, associated with amino acid metabolism and peptide biosynthesis respectively, were induced by microbial colonization (*P *< 0.05). Expression of the metallophosphoesterase-1 gene, whose product is involved in purine metabolism, was significantly upregulated, whereas expression of the thymidylate synthetase gene was downregulated (*P *< 0.05) in CONV versus GF animals. Overall, microbial colonization induced the expression of genes involved in nutrient transport and carbohydrate, protein, lipid and nucleotide metabolism.

### Electron transport

The expression of several genes involved in electron transport such as flavin containing monooxygenase-5 (*FMO5*), cytochrome P450, family 19, subfamily A, polypeptide 1 (*CYP19A1*), acyl-coenzyme A dehydrogenase (*ACADSB*), aldo-keto reductase family 1, member D1 (*AKR1D1*), thioredoxin domain containing I (*TXNDC1*) and cytochrome c oxidase (*COX8A*) was significantly upregulated in CONV versus GF animals (*P *< 0.05; Table 1). Microbial colonization downregulated expression of NADH dehydrogenase (ubiquinone) 1 beta subcomplex, 5 (*NDUFB5*), the product of which is associated with oxidative phosphorylation (*P *< 0.05; Table 1). These results demonstrate that microbiota induced genes involved in catalysis of various oxidative reactions.

### Immune responsiveness

Consistent with the previous observations [14,15], microbiota induced expression of the gene encoding bactericidal permeability-increasing protein (*BPI*), which damages bacterial inner/outer membranes and contributes to neutralization of bacterial lipopolysaccharide (LPS, Table 1). The complex of MHC class I α-chain, beta 2 microglobulin (B2M), transporter-1 ATP-binding cassette sub-family B (TAP1), tapasin (TAPBP), calreticulin, and Erp57 comprises the peptide-loading complex for MHC class I antigen processing [16]. These genes are characteristically induced in response to inflammatory cues and increased expression of swine leukocyte antigen classical class I (SLA class I), *B2M*, *TAP1 *and *TAPBP*, in the current study, likely reflects microbiota induced epithelial inflammation. Enhanced expression of *HSPA6 *(heat shock protein 70) in CONV animals further confirms this outcome as this gene is also induced by IFNβ [17].

### Identification of significantly enriched biological processes

**Figure 1:**
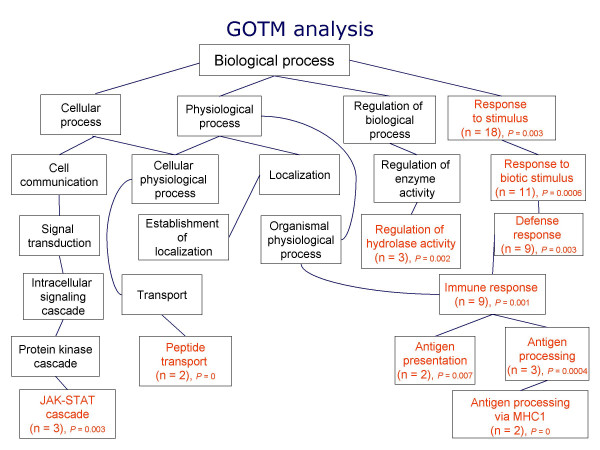
**Directed acyclic graph (DAG) of significantly enriched GO categories generated using GOTree Machine (GOTM) software**. Categories with significantly enriched gene numbers as determined by hypergeometric test are indicated in red while those in black are non-enriched. GOTM analysis demonstrated that more specific biological processes such as immune response, regulation of hydrolase activity, peptide transport and JAK-STAT cascade were significantly modulated by the microbiota. Number of observed genes in a particular biological process is indicated by "n".

GOTM analysis was performed to investigate whether certain biological processes or interactions were significantly enriched compared with all genes on the array. GO terms are connected into nodes of a network, thus the connections between 'parents' (broad/high level process) and 'children' (more specific/lower level process) are illustrated as directed acyclic graphs (Figure 1). The broad biological process "response to stimulus" and its child "response to biotic stimulus" followed by the more specific process "immune response" were significantly affected by the microbiota. The GO Consortium defines "response to biotic stress" as "a change in the state of an organism in the presence of a biotic stimulus, including response to bacteria, fungi, pest/pathogens/parasite" [18]. The ileal microbiota also significantly modulated three additional biological processes including the Janus activated kinase-STAT (JAK-STAT) cascade, peptide transport, and regulation of hydrolase activity. The specific genes comprised within these significant biological processes are listed in Additional file 5.

### Class prediction analysis

Class prediction analysis was performed using GeneSpring GX 7.3.1 (Agilent Technologies, Palo Alto, CA). All genes on the array were examined individually and ranked based on their power to discriminate GF versus CONV epithelia using cutoff points (*P *≤ 0.05; number of neighbors = 10; number of predictor genes = 20) and support vector machine algorithms. P-values were computed with Fischer's exact test to determine associations between state of microbial colonization (i.e. GF versus CONV) and normalized expression level for each gene. Thirteen of the 20 top-scoring predictive genes including *HLA-B, TAP1*, *B2M*, interferon-induced protein 44-like (*IFI44L*), and *STAT1 *were differentially expressed in CONV versus GF epithelia [see Additional file 6]. This outcome further supports the conclusion that microbial colonization activated the expression of STAT1 and the induction of interferon-inducible genes.

### Gene network interactions

**Figure 2:**
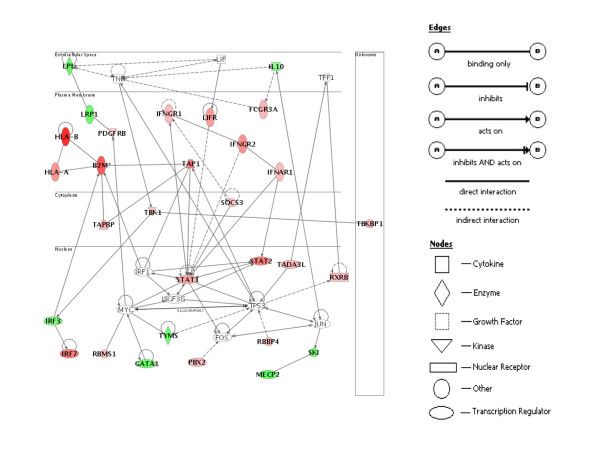
**Functional relationships among the differentially expressed genes were generated using Ingenuity Pathways Analysis**. Direct interactions are shown by solid lines and indirect interactions are depicted by broken lines. Red and green colors indicate up- and downregulation in CONV versus GF epithelia, respectively, whereas no color indicates that nodes were not differentially expressed on the array. The network is showing direct literature-supported relationships and confirms the interferon-mediated activation of transcription factors STAT1 and STAT2, which are involved in the expression of IFN-inducible target genes such as class I *MHC *(*HLA-B *in figure; pan SLA I on array and by qRT-PCR), *B2M*, *TAP1*, *TAPBP*, and *SOCS3 *in CONV compared with GF piglets.

Biological interactions among the selected genes were identified using Ingenuity Pathways Analysis software (Ingenuity Systems, Redwood City, CA) [19]. Among 223 differentially expressed genes, Ingenuity Pathways Analysis identified 112 genes contributing to a total of nine molecular networks. Each of networks 1 to 5 contains more than 10 genes, which are associated with immune response, cell growth and proliferation, DNA replication and recombination, cellular development and immune response, and cell death, respectively. Networks 1 to 5 were also interconnected and merged together to build a combined network representing the underlying biology (Figure 2). The composite network showed direct literature-supported relationships and further confirmed the IFN receptor-mediated activation of transcription factors STAT1 and STAT2, which are involved in the expression of IFN-inducible target genes such as *HLA-B*, *B2M*, *TAP1*, *TAPBP *and *SOCS3 *in CONV compared with GF animals. Therefore, for validation, we focused on this regulatory pathway along with Toll-like receptor (TLR)-mediated activation and regulation of immune responses by qRT-PCR.

### Candidate genes for qRT-PCR

Twenty three genes were chosen for two purposes: (I) to validate expression patterns of differentially expressed genes in the microarray studies, and (II) to examine possible regulatory mechanisms involved in microbiota-induced epithelial gene expression. First, eight genes (pan SLA I (primers do not distinguish SLA Ia and Ib transcripts), *B2M*, *HSPA6*, *SOCS3*, *IFNGR1*, *IFNAR1*, *LIFR*, *and STAT1*) that were differentially expressed according to microarray analysis were assayed by qRT-PCR. Second, the expression of fourteen non-differentially expressed (FDR *P *> 0.05) genes, which contribute to TLR and IFN receptor-mediated regulation of immune responses was determined by qRT-PCR. These targets included *TLR4, TLR9 *(which was not on the array), interleukin 10 (*IL10*), *IFNGR2*, myeloid differentiation protein-88(*MYD88*), *TOLLIP*, TRAF family member-associated NF-κB activator (TANK)-binding kinase-1 (*TBK1*), inhibitor of κB kinase epsilon (*IKBKE*), nuclear factor κB (*NFκB1*), NF-kappa-B inhibitor alpha (*NFκBIA*), IFN-regulatory factor 3 (*IRF3*), *IRF7*, *IFR9*, peroxisome-proliferator-activated receptor γ (*PPARG*), and *STAT2 *(Table 2).

**Table 2:** Comparison of expression patterns (fold change) observed with microarray versus qRT-PCR analysis

Gene symbol	Gene expression with qRT-PCR^1^				Microarray analysis	
	
	GF	CONV	Ratio	*P *value	Ratio	FDR adjusted *P *value
*NFκBIA*	1.00 ± 0.21	1.81 ± 0.21	1.81	0.05	1.79	*P *> 0.05
*TOLLIP*	1.00 ± 0.08	1.69 ± 0.12	1.69	0.01	0.97	*P *> 0.05
*IRF7*	1.00 ± 0.55	3.09 ± 0.63	3.09	0.05	0.83	*P *> 0.05
*IFNAR1*	0.73 ± 0.22	1.56 ± 0.22	2.14	0.04	1.53	0.013
*IFNGR1*	1.00 ± 0.32	2.62 ± 0.46	2.62	0.05	1.52	0.030
*LIFR*	0.52 ± 0.54	1.42 ± 0.63	2.73	0.07	2.31	0.003
*STAT1*	0.98 ± 0.28	2.09 ± 0.28	2.13	0.03	1.86	0.001
*STAT2*	1.00 ± 0.18	3.05 ± 0.20	3.05	0.03	1.01	*P *> 0.05
pan SLA I	1.42 ± 0.73	4.53 ± 0.85	3.19	0.04	2.44	0.001
*B2M*	1.13 ± 0.36	5.17 ± 0.42	4.58	0.01	3.97	0.009

^1^Values are means ± SE, n = 3/4 per treatment group. RNA was isolated from ileal sample of GF versus CONV piglets using Trizol reagent. Quantitative RT-PCR analysis of gene expression was determined by QuantiTect SYBR Green RT-PCR Master Mix in a 384-well plate using an ABI Prism 7900 HT SS instrument.

The mRNA expression patterns for the eight differentially expressed genes detected by microarray analysis were also differentially expressed when assayed by qRT-PCR confirming the microarray data. Expression patterns of 10 of 14 non-differentially expressed genes detected by microarray analysis were also similar when assayed by qRT-PCR. However, four genes (*NFκBIA*, *TOLLIP*, *IRF7*, and *STAT2*), which exhibited similar levels of expression on the array were significantly upregulated in CONV versus GF pigs when analyzed by qRT-PCR possibly indicating the higher sensitivity of qRT-PCR in detecting some transcripts or splice variation in transcripts [20]. Overall, RT-PCR results validate the microarray data and demonstrate good correlation between two assays.

## Discussion

### General

In the present study, the combination of LCM with microarray and qRT-PCR analyses was used to measure host gene expression profiles induced by the microbiota in the ileal epithelium of neonatal piglets, an *in vivo *model that preserves the contributions of other cell lineages and environmental factors in shaping the response. Differentially expressed genes involved in biological processes such as epithelial cell turnover, nutrient transport and metabolism, xenobiotic metabolism, JAK-STAT signaling pathway, and immune responsiveness were upregulated by the intestinal microbiota. Gene network analysis also revealed that microbial colonization activated both type I (IFNAR) and type II (IFNGR) interferon receptor mediated signaling cascades leading to enhanced expression of STAT1, STAT2 and IRF7 transcription factors and the induction of IFN-inducible genes as a reflection of intestinal epithelial inflammation. Enhanced expression of *NFκBIA *and *TOLLIP *and downregulation of *GATA1 *in colonized versus GF animals might reflect the activation of pathways that prevent excessive inflammation. Overall, the data are consistent with the hypothesis that the intestinal epithelium maintains a physiological state of inflammation, due to continuous microbial exposure, that results in a tight intestinal barrier, which serves to minimize antigen translocation to the lamina propria and unwarranted inflammatory responses to mutualistic microbes.

### Microbial induction of barrier function

Consistent with the maintenance of an intact mucosal barrier, thirteen genes contributing to cell proliferation, differentiation and regulation of cell growth were upregulated by bacterial colonization. In addition, expression of a gene involved in TNF receptor-mediated cell apoptosis was downregulated by resident microbiota [21]. Consistent with observations that goblet cell products provide innate protection that influences bacterial colonization [22,23], upregulation of beta 1,4-galactosyltransferase (*B4GALT1*) mRNA expression for mucin biosynthesis [24] in CONV piglets may reflect a general mucogenic response in conventionalized animals to maintain barrier function [22,25-27].

### Toll-like receptor-mediated signaling

The promotion of barrier function might be an inherent feature of mutualistic microbiota-induced TLR-mediated signaling [28,29]. In addition to the MyD88-dependent pathway that promotes rapid NFκB activation, TLRs also trigger MyD88-independent, IFN receptor-mediated signal transduction cascades upon the recognition of a variety of microbe-associated molecular patterns. These cascades activate members of the IRF family along with slow activation of NFκB [30]. The expression of *TLR4*, *TLR9*, *MYD88*, *TBK1*, *IKBKE*, *IRF3*, and *IRF9 *was unaffected but *IFNAR1*, *IFNGR1*, *and IRF7 *were induced by microbial colonization as determined by qRT-PCR analysis. A MyD88-independent pathway has been shown to activate IRFs and subsequently IFN-inducible genes in human embryonic kidney 293 cells [31], and mouse macrophages [32]. Similarly, mouse embryonic fibroblasts lacking MyD88 retained the ability to induce IFNα/β mRNA expression in response to viruses [33]. MyD88 is not required for the regulation of the majority of genes responsive to LPS and several MyD88-independent mouse genes contain NFκB and IFN-stimulated response element (ISRE; IFNα/β) binding sites [34]. MyD88-deficient mice also mount a normal host defense response to *Staphylococcus aureus *infection [35]. However, inflammatory responses during polymicrobial sepsis in mice deficient for MyD88 were markedly reduced implicating the importance of this signal transduction molecule in certain inflammatory scenarios [36]. The present study demonstrates that a MyD88-independent, IFN receptor-mediated pathway was activated by the intestinal microbiota in ileal epithelial cells of neonatal piglets, possibly reflecting a state of controlled inflammation. A potential limitation of the current study was the measurement of epithelial responses at a single time point 14 days after colonization. An early induction of TLR-mediated inflammation might have gradually become adapted to the continuous microbial exposure enabling a state of cellular homeostasis.

### IFN-mediated signaling

The data indicate that IFN receptor-mediated signaling cascades stimulated the expression of IFN-inducible genes in the ileal epithelium of CONV piglets. Interferon receptor complex IFNAR consists of two subunits IFNAR1 and IFNAR2, and IFNGR is comprised of IFNGR1 and IFNGR2 subunits [37]. Stimulation of IFNAR or IFNGR via the inflammatory cytokines IFNα/β and IFNγ activates receptor-associated Janus protein tyrosine kinases (Jak1 and Tyk2 for IFNAR and Jak1 and Jak2 for IFNGR) and the transcription factors STAT1 and STAT2 [38-40]. The phosphorylated STAT1/STAT2 heterodimers associate with IFR9 and bind to upstream regulatory consensus sequences of IFNα/β inducible genes (ISRE) [40]. In addition, formation of STAT1 homodimers stimulates transcription of genes containing IFNγ-activated sequence (GAS, cis-regulatory element) [40,41]. In the present study, microbiota activated IFN receptor-mediated signaling cascades possibly through *STAT1*, *STAT2 *and *IRF7 *transcription factors to induce IFN-inducible target genes such as pan SLA I, *B2M*, *HSPA6*, *TAP1*, *TAPBP*, *IFI44L *and *SOCS3 *in CONV compared with GF epithelia. Kawai et al. [42] demonstrated that the induction of IFNα/β mRNA was markedly inhibited in IRF7^-/- ^mouse embryonic fibroblasts and IRF7 activation was TBK1- and IKKε-independent in mouse plasmacytoid dendritic cells. Furthermore, Rhee et al. [43] reported that enteric *Salmonella *infection induced the expression of IFNγ regulated genes involved in antimicrobial defense in mice. IFNα/β signaling upregulates IFNγ production in T cells and dendritic cells [39,44]. Upregulation of *IFNAR1 *and *IFNGR1 *expression by the intestinal microbiota in the current study indicates that IFNα/β as well as IFNγ signaling pathways were activated in epithelial cells. The transcription factor STAT1 is common for both and crosstalk between these signaling pathways was reported by Takaoka et al. [39]. Therefore, STAT1 contributes to regulation of genes containing ISRE as well as GAS elements [45] and reciprocally affect each other's production and signaling [39]. Furthermore, SOCS3 is known to regulate the IFN signaling pathway by controlling STAT1 induction [46]. Overall, these results indicate that mutualistic microbes may activate IFN receptor-mediated signaling cascades in intestinal epithelial cells leading to activation of the transcription factors STAT1 and STAT2 and IFN-inducible genes harboring the cis-regulatory elements ISRE and GAS.

### Regulation of inflammatory processes

Resident microbiota can inhibit NFκB nuclear localization [47] and TLR4 induction [48] and thereby possibly prevent overt inflammatory responses. In the present study, microbial colonization induced *NFκBIA *expression, which encodes I-kappa-B-alpha, (IKBα), a protein associated with the inactivation of NFκB by sequestering it in the cytoplasm, consistent with inhibition of NFκB mediated inflammatory responses [47]. This outcome together with the induction of *TOLLIP *expression in CONV piglets possibly contributed to the suppression of an excessive inflammatory response to maintain intestinal homeostasis. TOLLIP inhibits TLR signaling by interfering with IL-1 receptor-associated kinase (IRAK) [49]. Indeed, intestinal epithelial cells express low levels of TLR4 and high levels of TOLLIP to prevent excessive inflammation [50].

The transcription factor GATA-1 upregulates TNFα-induced mRNA expression of chemokines eotaxin, RANTES and monocyte chemotactic protein-1 (MCP1) in airway epithelial cells [51]. Downregulation of *GATA1 *might have also contributed to intestinal homeostasis by inhibiting the expression of inflammatory chemokines. However, a comparatively low activation of NFκB along with steady-state activation of IFN receptor-mediated gene induction was maintained in the intestinal epithelium of animals harboring microbiota. This finding is consistent with an earlier report on the same model of an increased number of intraepithelial lymphocytes in the small intestine of CONV versus GF piglets [52].

This physiological state of inflammation was also associated with enhanced expression of genes associated with cell proliferation. NFκB is constitutively active in tumor cell lines derived from hematopoietic and solid tumors [53] and epithelial malignancies including colorectal, breast, lung, pancreas, and prostate cancers [54]. Suppression of NFκB in tumor samples inhibits proliferation, causes cell cycle arrest, and leads to apoptosis [55]. In addition to carcinoma cells, NFκB is also active in proliferating T cells, B cells, thymocytes, monocytes and astrocytes [53] indicating a crucial role of NFκB in cell proliferation. Furthermore, enhanced expression of LIFR in CONV piglets is consistent with involvement of its product in epithelial cell proliferation. LIF, a member of the inflammatory IL-6 cytokine family [56], induced proliferation of premalignant epithelial cells [57] and stimulates self-proliferation of embryonic stem cells [58].

## Conclusion

The present study indicates that microbial colonization transcriptionally induces the expression of cell membrane receptors and transcription factors, which are involved in the induction of IFN-inducible genes. Furthermore, overt inflammatory responses were possibly controlled through the induction of *NFκBIA *and *TOLLIP *and down-regulation of *GATA1 *expression, consistent with the maintenance of intestinal homeostasis. Collectively, the study supports the concept that the intestinal epithelium maintains a physiological state of inflammation with respect to continuous microbial exposure, which serves to maintain a tight intestinal barrier, without engendering overt inflammatory responses that would compromise barrier function.

## Methods

### Animals and experimental design

Two crossbred sows were purchased from Prairie Swine Center (Saskatoon, Canada) and housed in animal facilities until 113 d gestation. Eight cesarean-derived piglets from two litters were randomly assigned to two GF isolators (4 piglets per isolator) located at the University of Saskatchewan gnotobiotic animal facility. Using bottles fitted with nipples, all piglets were fed at 3 hr intervals to satiety a sterile porcine serum (Gibco, Burlington, Canada) and infant milk formula (Similac^®^, Abbott Laboratories, Abbott Park, IL) in 1:1 ratio. After 24 hrs, piglets were fed sterile infant formula (2:1 mixture of Similac and water) *ad libitum *from individual troughs replenished every 8 hrs for 14 d. Experimental protocol was reviewed and approved by the University of Illinois and the University of Saskatchewan, Institutional Animal Care and Use Committees, and was performed in accordance with recommendations of the Canadian Council on Animal Care.

### Microbial colonization and confirmation of GF status

Four piglets from one isolator were orally inoculated by adding 2 ml of the fecal slurry to the milk after 24 and 30 hrs postpartum. Four piglets in another isolator were kept GF throughout the study. Sterile swabs were wiped perianally daily during the experiment. The swabs were submerged in tubes of brain-heart infusion broth (Difco Laboratories, Sparks, MD) with 0.5% cysteine hydrochloride and were monitored for the development of turbidity. GF status of the piglets was confirmed by observing lack of microbial growth in the medium. An opaque color with obvious precipitate was observed in all tubes with swabs taken from CONV piglets indicating microbial colonization. Anaerobic and aerobic culture of fecal swabs collected throughout the experiment, together with the culture in blood agar base (BBL, Sparks, MD) with 5% defibrinated sheep blood for 48 hrs at 37°C of cecal digesta collected at the end of the experiment, further confirmed GF and CONV status. These procedures were described in detail by Shirkey et al. [52].

### Tissue collection and cryopreservation

Animals were euthanized by CO_2 _asphyxiation and exsanguination on d 14 and the small intestine was rapidly dissected and the length was measured. Two cm long segments starting at the 85% of SI length measured distally of the pyloric sphincter were identified, embedded in Shandon Cryomatrix™ (Thermo Electron Corporation, Pittsburgh, PA) and immediately frozen in liquid nitrogen. The samples were transported to the laboratory on dry ice and stored at -80°C until further analysis.

### Villus and crypt cell isolation by laser capture microdissection (LCM)

Laser capture microdissection was used to recover epithelial villus and crypt cells from frozen ileal sections. The cryostat (Leica CM 3050, Leica Microsystems, Deerfield, IL) was cleaned with 100% ethanol to avoid cross contamination, and a fresh disposable blade was used to cut each tissue. Frozen ileal tissue sections were placed in the cryostat for about 10 min to allow adjustment to the cutting temperature (-15 to -20°C). Tissue sections (8 μm thick) were cut and placed on Silane Prep slides (Sigma, St. Louis, MO) and stored at -80°C. A HistoGene™ staining kit (Arcturus, Mountain View, CA) was used to prepare the tissue for LCM. Briefly, the slides were thawed for 30 s, fixed in nuclease-free 75% ethanol, rehydrated in nuclease-free distilled water for 30 s, stained with HistoGene stain for 20 s, and rinsed in nuclease-free water for 30 s. Slides were then dehydrated by sequential immersion in a graded ethanol series (75%, 95% and 100% ethanol for 30 s each), followed by 5 min in xylene. Tissue sections were then dried in a slide box with desiccant for 5 min at room temperature before cell capture with LCM. Microdissection and capture of villus and crypt cell populations were performed immediately on a Pixcell II ^® ^LCM System (Arcturus) according to manufacturer instructions. A glass slide was mounted on the stage of the microscope and a CapSureTM LCM cap (Arcturus) was placed over the tissue and a low power infrared laser was pulsed to activate the transfer film and captured villus and crypt cells. Two hundred to five hundred cells were collected onto each cap using the following parameters: spot size, 7.5 μm; power, 50 mW; pulse duration 2.00 s.

### RNA isolation and amplification

Total RNA was isolated from laser captured villus and crypt cells using the PicoPure™ RNA isolation Kit (Arcturus) according to manufacturer recommendations. Briefly, 20 μl of extraction buffer was placed into the LCM assembly microcentrifuge tube and incubated for 30 min at 42°C. After incubation, the assembly was briefly centrifuged to collect the extraction fluid into the microcentrifuge tube. The fluid was then loaded onto a spin column, washed several times, and the total cellular RNA was eluted as 10 μl volume. RNA from each sample was subjected to 2 rounds of amplification using the RiboAmp™ RNA linear amplification kit (Arcturus). Subsequently, RNA was reverse-transcribed into cDNA incorporating a T7 promoter. The cDNA was eluted with 16 μl of elution buffer and in vitro transcribed into amplified anti-sense RNA with a T7 RNA polymerase incorporating amino-allyl-UTP (Ambion, Austin, TX).

### Probe labeling for microarray analysis

The amplified anti-sense (aRNA) was labeled with Cy3 or Cy5 dyes. Each of the 16 cell populations was reversed labeled with Cy3 and Cy5 to account for dye labeling bias, resulting in 32 target samples for microarray hybridization. Cy3 and Cy5 dyes were resuspended in 45 μl dimethyl sulfoxide (DMSO, Sigma) and stored at -80°C in a light protected area. For the labeling reaction, 5–7 μg of amino-allyl tagged aRNA was dried in a SpeedVac and 5 μl of 0.1 M Na_2_CO_3 _(pH, 9.0) and 5 μl of dye (Cy3 or Cy5) dissolved in DMSO were added to the dried sample. The mixture was then incubated in the dark for 75 min and unincorporated dye was removed with a modified protocol based on the Qiagen Mini Elute Kit (Qiagen, Valencia, CA) with a total elution volume of 50 μl for each sample. The sample was then dried in a SpeedVac for 50 min to dryness and resuspended in 10 μl of RNase free water. To decrease the fragment size to 60–200 bases, 1 μL of fragmentation buffer (Ambion) was added to the sample and heated to 70°C for 15 min. Stop solution (1 μl) was added to terminate the reaction. A blocking mixture containing 2 μl of poly d(A) and 20 μl of Porcine Hybloc™ (Applied Genetics Laboratories, Melbourne, FL) were added to 12 μl probe and incubated at 100°C for 1 min then snap-cooled on ice for 2 min. Subsequently, the mixture was centrifuged to dryness in a SpeedVac and 80 μl of the hybridization buffer (20% formamide, 5× Denhardts, 6× SSC, 0.1% SDS, 25 μg/ml tRNA, and RNase free water) was added to each dried sample. The probe was warmed to 42°C for 5 min and maintained at this temperature until hybridization.

### Microarray development and hybridization

Details on the design and validation of the porcine oligonucleotide set have been reported [9]. This set represents 13,297 porcine cDNAs and ESTs. A reference design was used in which each of the experimental samples was cohybridized with the reference sample that contained equal amounts of all the RNA samples used in the experiment, which allowed the treatment of fluorescence ratios as measurements of relative expression. Single-spotted oligos were printed on GAPS II slides (Corning, Corning, NY) at the W. M. Keck Center for Comparative and Functional Genomics, University of Illinois. Before hybridization, the arrays were plunged in 0.2% SDS water and immediately agitated vigorously for 2 min, washed three times in successive jars of distilled water and rinsed in 100% ethanol. The arrays were then incubated in 5× SSC, 0.1% SDS, 1% BSA in a Coplin jar for 45 to 60 min at 42°C, washed by immersing in 5 successive Coplin jars of deionized distilled water (RICCA Chemical Company, Arlington, TX), rinsed in 100% isopropanol, to ensure that the SDS was completely removed from the arrays and subsequently dried by centrifugation. An array was placed into a Corning Hybridization Chamber (Sigma) and 80 μl of the labeled solution was applied to a slide and covered with a LifterSlip™ (Erie Scientific, Portsmouth, NH). The chamber was then assembled and submerged in a 42°C water bath for 48 hrs.

### Post-hybridization wash and array scanning

The hybridization chamber was disassembled and the LifterSlip™ was removed by immersing the array in 2× SSC, 0.1% SDS (at 42°C) until the LifterSlip™ moved freely away from the slide. The arrays were placed in 2× SSC, 0.1% SDS for 5 min at 42°C. Subsequently the arrays were placed in 0.1× SSC, 0.1% SDS for 10 min at room temperature followed by 0.1× SSC for 1 min at room temperature. The arrays were then rinsed in 0.01× SSC for 10 sec or less and dried by centrifugation. Following hybridization and washing, slides were scanned immediately for both dye channels with an Axon 4000B (Molecular Devices, Union City, CA) dual-laser confocal scanner and images were processed using GenePix v6.0 software (Molecular Devices). Microarray data from this study are available at The National Center for Biotechnology Information (NCBI) Gene Expression Omnibus (GEO) database (GSE7232) [59].

### Microarray performance and data analysis

During an initial screening, microarrays that did not contain at least 50% of the total number of spots with median background-subtracted signal intensities >3 SD above background in both Cy3 and Cy5 channels were repeated. Data from a total of 32 microarrays were normalized and used for statistical analysis. Median foreground intensities (F) and median background intensities (B) were generated to construct the data matrix. Data were filtered out when the following criteria were satisfied: (I) removal of low intensity spots when F-B < M + 2 MAD, where M is the median of the negative control spots (blank or buffer spots); MAD is the median absolute deviation, (II) removal of saturated spots with very high intensity, and (III) removal of spots flagged by GenePix. Data were normalized using the regional lowess normalization (rlowess) method from the MAANOVA package [60] and analyzed by Proc GLM in SAS (version 8.2). The data was fitted to the model: *y*_*ijklm *_= *μ *+ *A*_*i *_+ *D*_*j *_+ *T*_*k *_+ *C*_*l *_+ *T*_*Ckl *_+ *S*_*m *_+ *e*_*ijklm *_where *y*_*ijklm *_is the logarithm of signal intensity, *μ *is the overall mean expression level, *A*_*i *_is the effect of the *i *th array, *D*_*j *_is the effect of the *j *th dye, *T*_*k *_is the effect of the *k *th treatment, *C*_*l *_is the effect of the *l *th tissue, *TC*_*kl *_is the interaction effect of the *k *th treatment and the *l *th tissue, *S*_*m *_is the *m *th sample effect, and *e*_*ijklm *_is random effect and is assumed to follow normal distribution with 0 mean and constant variance. To avoid type I error of least square means, Proc multtest was conducted to generate a false discovery rate (FDR) value [61]. FDR adjustments balance type I and type II error rates. Genes were considered to be differentially expressed when FDR adjusted *P *= 0.05.

### Microarray annotation

Basic Local Alignment Search Tool (BLASTN) analysis against 13,297 spotted oligonucleotides was performed against tentative consensus (TC) sequences from The Institute for Genomic Research (TIGR) database [63] (build 12; 42 sequences) and porcine UniGene database from the NCBI database [64] (build 27; 544 sequences) to identify swine-specific genes, EST clusters, and annotations. In addition, BLASTN for all sequences was performed against human UniGene (build 199; 727 sequences), mouse UniGene (build 161; 13 sequences), mouse mRNA (236 sequences), and human mRNA sequences (11,717 sequences) using an E-value cut-off of E ≤ e-5 and an extension threshold of 40 (NCBI February 28, 2007) [64]. For all searches, best hits were used to annotate the swine sequences. A total of 10,560 oligos had GO annotation, and they resulted in 6,344 different NCBI gene IDs. GO terms were obtained (February 28, 2007) from the GO database [65]. Perl scripts were used to annotate the porcine sequences with relevant information parsed from human UniGene and LocusLink (e.g., gene symbol, gene name, function, OMIM number, PubMed identification numbers) to obtain GO annotations associated with human and mouse UniGene numbers.

### Approach for data mining

First, principal component analysis (PCA) was used to identify components having the greatest effect on variance in gene expression. Second, genes that were differentially expressed were categorized by their involvement in GO biological processes. Third, GOTree Machine (GOTM) analysis was used to identify GO categories with relatively enriched gene numbers that were significantly impacted by microbial state (GF versus CONV). Fourth, class prediction analysis was performed to predict marker genes in CONV versus GF epithelia. Fifth, biological interactions among differentially expressed genes were identified using Ingenuity Pathways Analysis 3.1 software [19]. Finally, quantitative RT-PCR was used to validate the microarray results for pathways selected by GOTM and Ingenuity Pathways Analysis.

### Principal component analysis (PCA)

Differentially expressed genes were subjected to PCA using GeneSpring GX 7.3.1 (Agilent Technologies) to determine patterns in the variability of expression profiles. PCA is a mathematical method that reduces the number of dimensions in a large dataset to a few dimensions that explain the majority of the variation between samples [62].

### Gene ontology, class prediction and biological pathways analyses

Differentially expressed genes were categorized according to their involvement in GO biological processes. Furthermore, GOTM analysis was performed to identify specific biological processes (i.e., number of genes operative in the process) that were significantly enriched among the differentially expressed genes relative to their abundance on the microarray [66]. Class prediction analysis was conducted via GeneSpring GX 7.3.1 (Agilent) to predict marker genes for microbe-activated intestinal epithelial cells. Finally, functional relationships among the differentially expressed genes were generated using Ingenuity Pathways Analysis software.

### Quantitative real-time RT-PCR (qRT-PCR)

Total RNA was isolated from ileal samples using TRIZOL reagent (Invitrogen, Carlsbad, CA) for qRT-PCR to validate microbial colonization-induced changes in the expression of genes involved in selected pathways. RNA was purified with the RNeasy Mini kit and residual DNA removed using the RNase-Free DNase set (Qiagen). RNA quality was determined to check the integrity of 28S and 18S rRNA using Agilent 2100 Bioanalyzer (Agilent) and total RNA yield was quantified by spectrophotometry. Porcine-gene-specific primers were designed by Primer Express Software v2.0 (Applied Biosystems, Foster City, CA) and PRIMER3 algorithm in Biology WorkBench [67] [see Additional file 7]. One step RT-PCR was performed using Qiagen reagent (Qiagen) according to the manufacturer's instructions and a GeneAmp PCR System 2700 thermocycler (Applied Biosystems) to confirm single product amplification. Quantitative RT-PCR reactions were performed using SYBRGreen Master Mix according to the manufacturer's instructions (Qiagen). The relative expression of mRNA transcripts was measured in triplicate in a 384-well plate using an ABI Prism 7900 HT SS instrument (Applied Biosystems) after normalization to 18S rRNA. Data were analyzed by SAS and *P *< 0.05 was considered significant.

## Authors' contributions

SRC carried out the qRT-PCR analysis, coordinated the bioinformatic analysis of the microarray and qRT-PCR data, and drafted the manuscript along with HRG. DEK participated in epithelial sample collection, RNA isolation, microarray hybridization and drafted an earlier version of the manuscript. BPW conducted the animal trial. MRB, ABL, LAR, and JEB assisted with either the laser capture microdissection or microarray hybridization and interpretation of data. JCM designed the statistical models used for data analysis. JJL contributed to the bioinformatic analysis of the microarray and qRT-PCR data and interpretation of the results. LBS, AGVK and HRG conceived and coordinated the study. All authors read and approved the final manuscript.

## Supplementary Material

Additional file 1 Principal component analysis. Principal component analysis (PCA) was performed using GeneSpring software (Agilent) to reduce the number of variables in the multivariate data. Click here for file

Additional file 2 Expression profiles of genes associated with various biological processes in CONV compared with GF crypts. Table lists the differentially expressed genes involved in transcription, signal transduction, cell proliferation and differentiation, metabolism, electron transport, immune response and other biological processes in ileal epithelia from crypts of conventional versus germfree animals. Click here for file

Additional file 3 Expression profiles of genes associated with various biological processes in CONV compared with GF villi. Table lists the differentially expressed genes involved in immune response and other biological processes in ileal epithelia from villi of conventional versus germfree animals. Click here for file

Additional file 4 The differentially expressed genes were categorized according to GO biological processes. A pie chart depicts the percentage of differentially expressed genes in the GO biological processes of transcription, signal transduction, cell cycle, transport, metabolism, immune response, electron transport, others, and unknown. Click here for file

Additional file 5 Genes comprising the enriched biological processes identified by GOTM analysis. Table lists the differentially expressed genes associated with significantly enriched biological processes as determined by GOTM analysis. Click here for file

Additional file 6 Marker genes identified by class prediction analysis. Table lists marker genes that distinguish germfree versus conventional ileal epithelia identified by class prediction analysis with GeneSpring software (Agilent). Click here for file

Additional file 7 Sequences for qRT-PCR primers. Table lists the porcine-gene-specific primers used for qRT-PCR analysis. Click here for file
