# Supplementary material for: Transcriptome profiling of the small intestinal epithelium in germfree versus conventional piglets
Source: BMC Genomics. 2007 Jul 5;8:215. doi: 10.1186/1471-2164-8-215 (PMC1949829; doi:10.1186/1471-2164-8-215)
Supplement: Additional file 1 — Principal component analysis. Principal component analysis (PCA) was performed using GeneSpring software (Agilent) to reduce the number of variables in the multivariate data. [file 1471-2164-8-215-S1.ppt]

## Slide 1
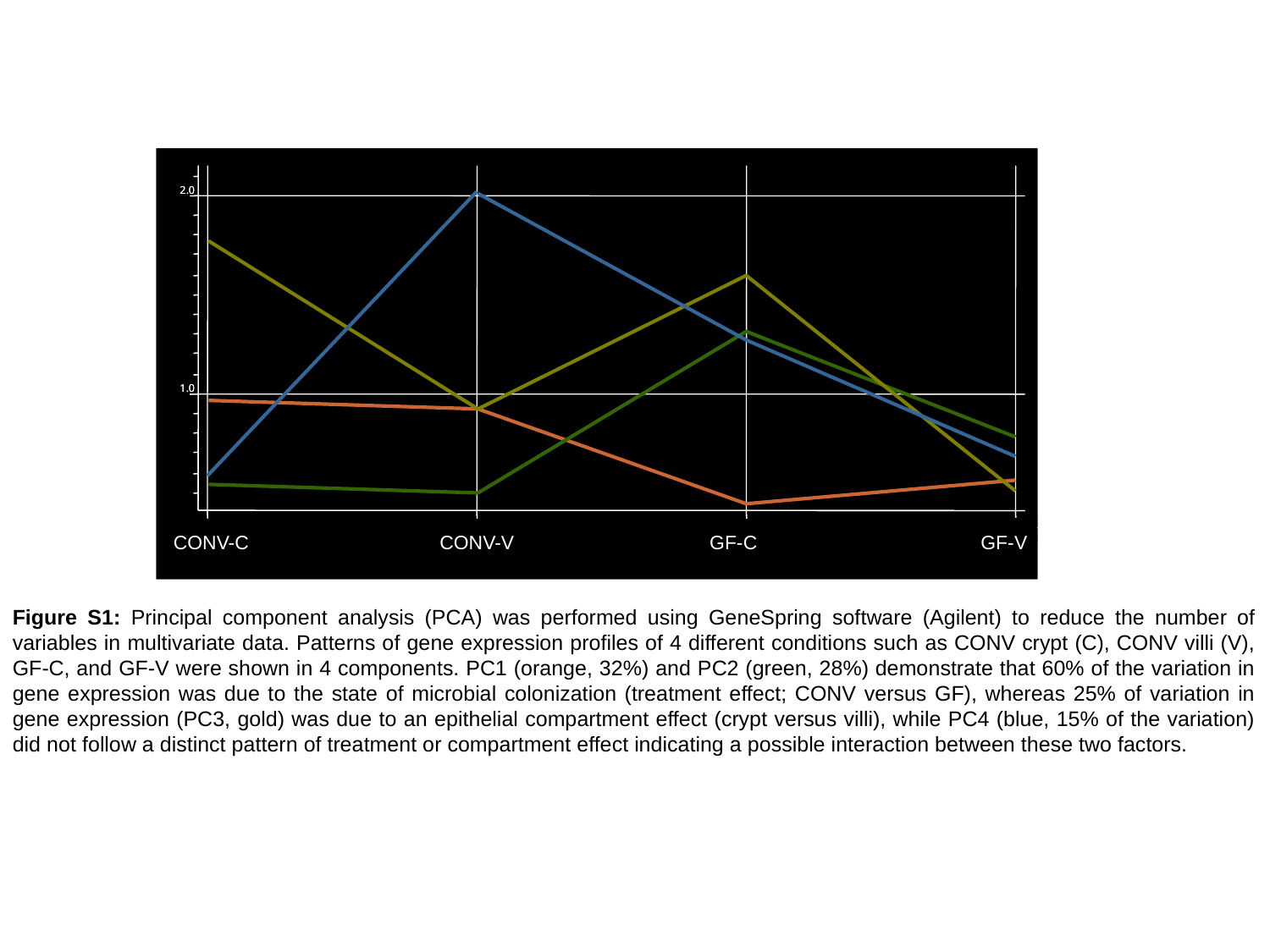

2.0
2.0
1.0
1.0
conv crypt
conv crypt
conv villus
conv villus
free crypt
free crypt
free villus
free villus
0
CONV-C
GF-V
CONV-V
GF-C
Figure S1: Principal component analysis (PCA) was performed using GeneSpring software (Agilent) to reduce the number of variables in multivariate data. Patterns of gene expression profiles of 4 different conditions such as CONV crypt (C), CONV villi (V), GF-C, and GF-V were shown in 4 components. PC1 (orange, 32%) and PC2 (green, 28%) demonstrate that 60% of the variation in gene expression was due to the state of microbial colonization (treatment effect; CONV versus GF), whereas 25% of variation in gene expression (PC3, gold) was due to an epithelial compartment effect (crypt versus villi), while PC4 (blue, 15% of the variation) did not follow a distinct pattern of treatment or compartment effect indicating a possible interaction between these two factors.
