# Supplementary material for: Transcriptome profiling of the small intestinal epithelium in germfree versus conventional piglets
Source: BMC Genomics. 2007 Jul 5;8:215. doi: 10.1186/1471-2164-8-215 (PMC1949829; doi:10.1186/1471-2164-8-215)
Supplement: Additional file 2 — Expression profiles of genes associated with various biological processes in CONV compared with GF crypts. Table lists the differentially expressed genes involved in transcription, signal transduction, cell proliferation and differentiation, metabolism, electron transport, immune response and other biological processes in ileal epithelia from crypts of conventional versus germfree animals. [file 1471-2164-8-215-S2.doc]

| Functional class | Unigene ID | Gene description | FDR adjusted *P* value | Fold change^1^ |
| --- | --- | --- | --- | --- |
| Transcription | Hs.524530 | CTD (carboxy-terminal domain, RNA polymerase II) small phosphatase 2 (*CTDSP2*) | 0.007 | 1.75 |
|  | Hs.444468 | CTD small phosphatase 1 (*CTDSP1*) | 0.007 | 1.79 |
|  | Hs.765 | GATA binding protein 1 (globin transcription factor 1, *GATA1*) | 0.006 | 0.65 |
|  | Hs.509545 | Pre-B-cell leukemia transcription factor 2 (*PBX2*) | 0.007 | 1.63 |
|  | Hs.433343 | Serine/arginine repetitive matrix 2 (*SRRM2*) | 0.001 | 1.81 |
|  | Hs.462379 | Target of myb1-like 2 (*TOM1L2*) | 0.004 | 1.67 |
|  | Hs.389734 | Transcription elongation factor A (SII)-like 8 (*TCEAL8*) | 0.007 | 0.56 |
|  | Hs.517296 | V-ets erythroblastosis virus E26 oncogene homolog 2 (*ETS2)* | 0.002 | 1.53 |
|  | Hs.490504 | Zinc finger protein 398 (*ZNF398*) | 0.017 | 1.62 |
|  | Hs.379548 | Zinc finger protein 650 (*ZNF650*) | 0.043 | 0.52 |
| Signal | Hs.371240 | A kinase (PRKA) anchor protein (gravin) 12 (*AKAP12*) | 0.031 | 1.94 |
| transduction | Hs.150423 | Cyclin-dependent kinase 9 (*CDK9*) | 0.039 | 1.59 |
|  | Hs.433879 | DNA directed RNA polymerase II polypeptide J-related gene (*POLR2J2*) | 0.001 | 1.46 |
|  | Hs.765 | GATA binding protein 1 (globin transcription factor 1, *GATA1)* | 0.006 | 0.65 |
|  | Hs.520019 | Mitochondrial coiled-coil domain 1 (*MCCD1*) | 0.010 | 1.84 |
|  | Hs.470943 | Signal transducer and activator of transcription 1 (*STAT1*) | 0.001 | 1.93 |
|  | Hs.512619 | Thymidine kinase 2, mitochondrial (*TK2*) | 0.042 | 1.57 |
|  | Hs.47061 | Unc-51-like kinase 1 (*C. elegans*) (*ULK1*) | 0.040 | 1.78 |
|  | Hs.469244 | WAS protein family, member 2 (*WASF2*) | 0.032 | 2.29 |
| Cell | Hs.130316 | Drebrin 1 (*DBN1*) | 0.045 | 1.53 |
| proliferation, | Hs.516633 | NCK-associated protein 1 (*NCKAP1*) | 0.013 | 0.66 |
| differentiation | Hs.191346 | Septin 7 (*SEPT7*) | 0.024 | 0.61 |
|  | Hs.530477 | Signal-induced proliferation-associated gene 1 (*SIPA1L1*) | 0.001 | 1.63 |
| Metabolism | Hs.81934 | Acyl-Coenzyme A dehydrogenase (*ACADSB*) | 0.043 | 2.06 |
|  | Hs.548558 | Cathepsin D (*CTSD*) | 0.034 | 1.55 |
|  | Hs.443161 | Dihydropyrimidinase (*DPYS*) | 0.047 | 0.43 |
|  | Hs.403436 | Dodecenoyl-Coenzyme A delta isomerase  (3,2 trans-enoyl-Coenzyme A isomerase, *DCI)* | 0.031 | 1.85 |
|  | Hs.26770 | Fatty acid binding protein 7 (*FABP7*) | 0.011 | 0.55 |
|  | Hs.418795 | Glycosyltransferase 25 domain containing 1 (*GLT25D1*) | 0.043 | 0.61 |
|  | Hs.512841 | Heparan sulfate 6-O-sulfotransferase 1 (*HS6ST1*) | 0.038 | 0.31 |
|  | Hs.180878 | Lipoprotein lipase *(LPL*) | 0.002 | 0.53 |
|  | Hs.368960 | N-glycanase 1 (*NGLY1*) | 0.043 | 1.71 |
|  | Hs.494261 | Phosphoserine aminotransferase 1 (*PSAT1*) | 0.043 | 1.64 |
|  | Hs.503251 | Protein phosphatase methylesterase-1 (*PME-1*) | 0.044 | 2.75 |
| Electron | Hs.156668 | Similar to B15 subunit of the NADH: ubiquinone oxidoreductase ([*LOC205243*](http://www.ncbi.nlm.nih.gov/entrez/query.fcgi?db=gene&cmd=Retrieve&dopt=full_report&list_uids=205243)) | 0.031 | 2.35 |
| transport | Hs.137335 | Hypothetical protein LOC285690 (*LOC285690*) | 0.039 | 2.24 |
|  | Hs.518424 | NADH dehydrogenase (ubiquinone) 1 beta subcomplex, 5, 16kDa (*NDUFB5*) | 0.008 | 0.36 |
| Immune | Hs.529019 | Bactericidal/permeability-increasing protein (*BPI)* | 0.002 | 2.01 |
| response | Hs.278694 | CD209 molecule (*CD209*) | 0.003 | 1.52 |
|  | Hs.3268 | Heat shock 70kDa protein (HSP70B*,* *HSPA6* ) | 0.001 | 1.28 |
|  | Hs.549047 | Immunoglobulin lambda constant 1 (Mcg marker) (*IGLC1*) | 0.001 | 4.23 |
|  | Hs.389724 | Interferon-induced protein 44-like (*IFI44L)* | 0.001 | 2.52 |
|  | [Hs.77961](http://www.ncbi.nlm.nih.gov/UniGene/clust.cgi?ORG=Hs&CID=77961) | Major histocompatibility complex, class I, B (*HLA-B*) | 0.001 | 2.68 |
|  | Hs.272011 | UDP-Gal:betaGlcNAc beta 1,4- galactosyltransferase, polypeptide 1 (*B4GALT1*) | 0.010 | 1.57 |
| Others | Hs.516468 | ADP-ribosylation-like factor 6 interacting protein 6 (*ARL6IP6*) | 0.043 | 2.91 |
|  | Hs.518059 | AER61 glycosyltransferase (*AER61*) | 0.043 | 0.57 |
|  | Hs.73708 | Ankyrin repeat domain 2 (stretch responsive muscle) (*ANKRD2*) | 0.043 | 0.68 |
|  | Hs.535257 | Centaurin, beta 5 (*CENTB5*) | 0.042 | 1.39 |
|  | Hs.470457 | COBL-like 1 (*COBLL1*) | 0.043 | 1.73 |
|  | Hs.239666 | Core 1 synthase, glycoprotein-N-acetylgalactosamine  3-beta-galactosyltransferase1 (*C1GALT1*) | 0.004 | 2.07 |
|  | Hs.509226 | FK506 binding protein 3, 25kDa (*FKBP3*) | 0.030 | 0.52 |
|  | Hs.497573 | FLJ45244 protein (*FLJ45244*) | 0.025 | 1.87 |
|  | Hs.211046 | Hypothetical protein LOC126248 (*LOC126248*) | 0.003 | 3.25 |
|  | Hs.371794 | KIAA1404 protein (*KIAA1404)* | 0.031 | 1.84 |
|  | Hs.381099 | Lymphocyte cytosolic protein 1 (L-plastin*, LCP1*) | 0.042 | 2.18 |
|  | Hs.514373 | Myotubularin related protein 4 (*MTMR4*) | 0.043 | 2.22 |
|  | Hs.527971 | Nestin (*NES*) | 0.032 | 1.74 |
|  | Hs.464184 | SEC14-like 1 (*S. cerevisiae*) (*SEC14L1*) | 0.048 | 1.77 |
|  | Hs.12102 | Sorting nexin 3 (*SNX3*) | 0.031 | 0.50 |

**Table S1: Expression profiles of genes associated with various biological processes in CONV**

**compared with GF crypts**

^1^Fold change is the ratio of CONV versus GF.
