# Supplementary material for: Transcriptome profiling of the small intestinal epithelium in germfree versus conventional piglets
Source: BMC Genomics. 2007 Jul 5;8:215. doi: 10.1186/1471-2164-8-215 (PMC1949829; doi:10.1186/1471-2164-8-215)
Supplement: Additional file 3 — Expression profiles of genes associated with various biological processes in CONV compared with GF villi. Table lists the differentially expressed genes involved in immune response and other biological processes in ileal epithelia from villi of conventional versus germfree animals. [file 1471-2164-8-215-S3.doc]

**Table S2: Expression profiles of genes associated with various biological processes**

**in CONV compared with GF villi**

| Functional class | Unigene ID | Gene description | FDR adjusted *P* value | Fold change^1^ |
| --- | --- | --- | --- | --- |
| Immune response | Hs.497573 | FLJ45244 protein (*FLJ45244*) | 0.025 | 2.18 |
|  | Hs.3268 | Heat shock 70kDa protein 6 (HSP70B, *HSPA6*) | 0.001 | 1.22 |
|  | Hs.389724 | Interferon-induced protein 44-like (*IFI44L*) | 0.001 | 2.53 |
|  | [Hs.77961](http://www.ncbi.nlm.nih.gov/UniGene/clust.cgi?ORG=Hs&CID=77961) | Major histocompatibility complex, class I, B (*HLA-B*) | 0.001 | 2.22 |
|  | Hs.13854 | T-cell activation protein phosphatase 2C (*PPTC7*) | 0.018 | 2.49 |
|  | Hs.352018 | Transporter 1, ATP-binding cassette, sub-family B (*TAP1*) | 0.001 | 4.01 |
| Others | Hs.443625 | Collagen, type III, alpha 1 (*COL3A1*) | 0.020 | 1.21 |
|  | Hs.524530 | CTD (carboxy-terminal domain, RNA polymerase II)  small phosphatase 2 (*CTDSP2*) | 0.007 | 2.37 |
|  | Hs.368011 | Mitochondrial ribosome recycling factor (*MRRF*) | 0.005 | 1.50 |
|  | Hs.514373 | Myotubularin related protein 4 (*MTMR4*) | 0.043 | 2.81 |
|  | Hs.527971 | Nestin (*NES*) | 0.032 | 2.03 |
|  | Hs.525091 | Testis-specific kinase 2 (*TESK2*) | 0.006 | 1.37 |

^1^Fold change is the ratio of CONV versus GF.
