# Supplementary material for: Transcriptome profiling of the small intestinal epithelium in germfree versus conventional piglets
Source: BMC Genomics. 2007 Jul 5;8:215. doi: 10.1186/1471-2164-8-215 (PMC1949829; doi:10.1186/1471-2164-8-215)
Supplement: Additional file 4 — The differentially expressed genes were categorized according to GO biological processes. A pie chart depicts the percentage of differentially expressed genes in the GO biological processes of transcription, signal transduction, cell cycle, transport, metabolism, immune response, electron transport, others, and unknown. [file 1471-2164-8-215-S4.ppt]

## Slide 1
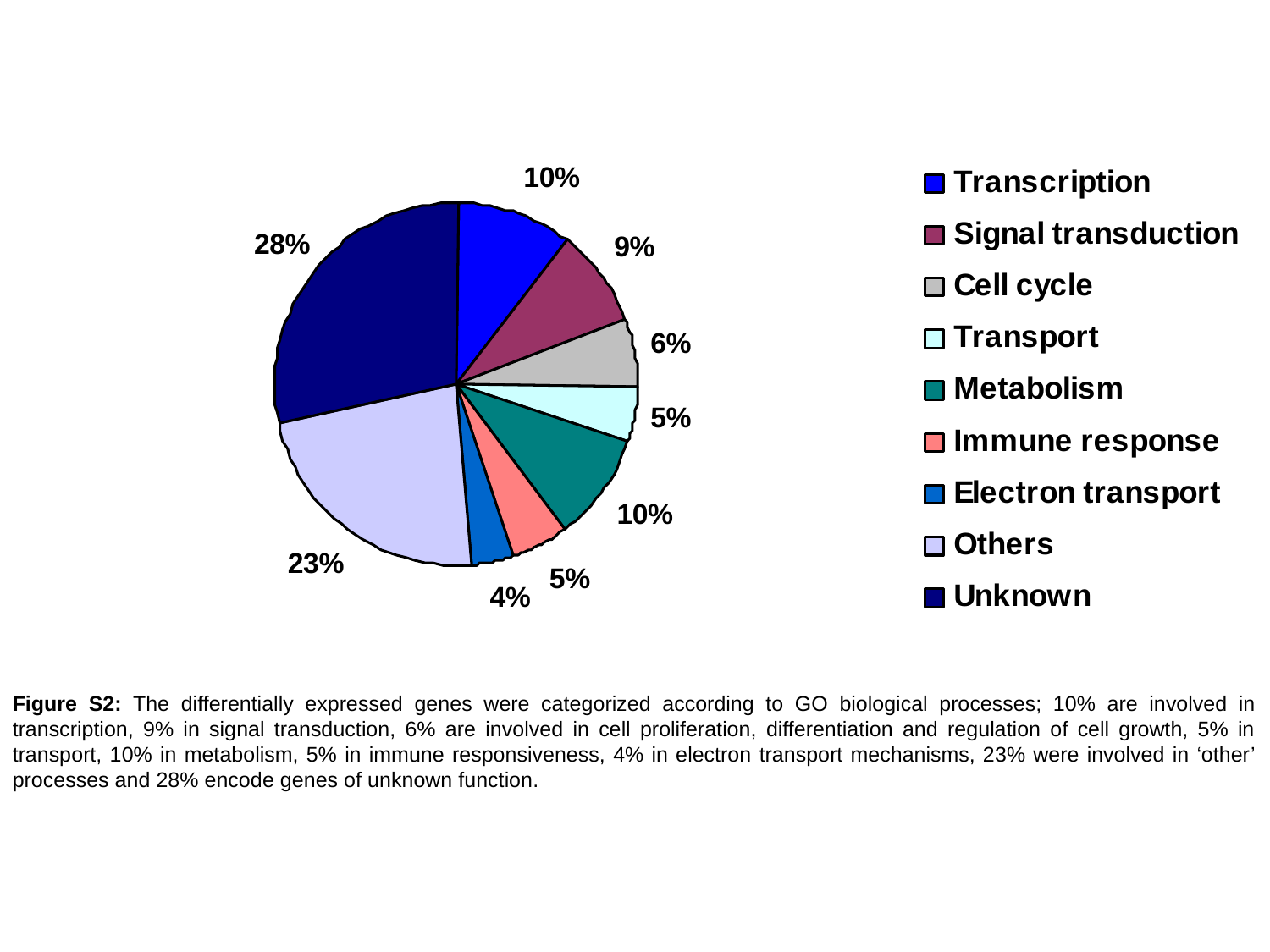

Figure S2: The differentially expressed genes were categorized according to GO biological processes; 10% are involved in transcription, 9% in signal transduction, 6% are involved in cell proliferation, differentiation and regulation of cell growth, 5% in transport, 10% in metabolism, 5% in immune responsiveness, 4% in electron transport mechanisms, 23% were involved in ‘other’ processes and 28% encode genes of unknown function.
