# Supplementary material for: Transcriptome profiling of the small intestinal epithelium in germfree versus conventional piglets
Source: BMC Genomics. 2007 Jul 5;8:215. doi: 10.1186/1471-2164-8-215 (PMC1949829; doi:10.1186/1471-2164-8-215)
Supplement: Additional file 5 — Genes comprising the enriched biological processes identified by GOTM analysis. Table lists the differentially expressed genes associated with significantly enriched biological processes as determined by GOTM analysis. [file 1471-2164-8-215-S5.doc]

**Table S3:** Genes comprising the enriched biological processes identified by GOTM analysis

| Functional class | Unigene ID | Gene description |
| --- | --- | --- |
| Response to | Hs.529019 | Bactericidal/permeability-increasing protein (*BPI*) |
| stimulus | Hs.534255 | Beta-2-microglobulin (*B2M*) |
|  | Hs.510402 | CD46 molecule, complement regulatory protein (*CD46*) |
|  | Hs.504641 | CD163 antigen (*CD163*) |
|  | Hs.278694 | CD209 antigen (*CD209*) |
|  | Hs.372679 | Fc fragment of IgG, low affinity IIIb, receptor (CD16b) (*FCGR3B*) |
|  | Hs.2006 | Glutathione S-tranferase M3 (*GSTM3*) |
|  | Hs.3268 | Heat shock 70kDa protein (HSP70B*,* *HSPA6* ) |
|  | Hs.648398 | Immunoglobulin heavy constant alpha 1 (*IGHA1*) |
|  | Hs.52940 | Interferon (alpha, beta and omega) receptor 1 *(IFNAR1*) |
|  | Hs.389724 | Interferon-induced protein 44-like (*IFI44L*) |
|  | Hs.77961 | Major histocompatibility complex, class I, B (*HLA-B*) |
|  | Hs.533282 | Non-POU domain containing, octamer-binding (*NONO*) |
|  | Hs.194756 | Sine oculis homeobox homolog 6 (Drosophila) (*SIX6*) |
|  | Hs.470943 | Signal transducer and activator of transcription 1 (*STAT1*) |
|  | Hs.370937 | TAP binding protein (tapasin, *TAPBP*) |
|  | Hs.495656 | Transducin (beta)-like 1X-linked (*TBL1X*) |
|  | Hs.352018 | Transporter 1, ATP-binding cassette, sub-family B (MDR/TAP) *TAP1* |
| Response to biotic | Hs.529019 | Bactericidal/permeability-increasing protein (*BPI*) |
| stimulus | Hs.510402 | CD46 molecule, complement regulatory protein (*CD46*) |
|  | Hs.504641 | CD163 antigen (*CD163*) |
|  | Hs.278694 | CD209 antigen (*CD209*) |
|  | Hs.372679 | Fc fragment of IgG, low affinity IIIb, receptor (CD16b) (*FCGR3B*) |
|  | Hs.648398 | Immunoglobulin heavy constant alpha 1 (*IGHA1*) |
|  | Hs.529400 | Interferon (alpha, beta and omega) receptor 1*(IFNAR1*) |
|  | Hs.77961 | Major histocompatibility complex, class I, B (*HLA-B*) |
|  | Hs.470943 | Signal transducer and activator of transcription 1 (*STAT1*) |
|  | Hs.370937 | TAP binding protein (tapasin, *TAPBP*) |
|  | Hs.352018 | Transporter 1, ATP-binding cassette, sub-family B (MDR/TAP) *TAP1* |
| Defense response/ | Hs.529019 | Bactericidal/permeability-increasing protein (*BPI*) |
| immune response | Hs.510402 | CD46 molecule, complement regulatory protein (*CD46*) |
|  | Hs.504641 | CD163 antigen (*CD163*) |
|  | Hs.278694 | CD209 antigen (*CD209*) |
|  | Hs.372679 | Fc fragment of IgG, low affinity IIIb, receptor (CD16b) (*FCGR3B*) |
|  | Hs.648398 | Immunoglobulin heavy constant alpha 1 (*IGHA1*) |
|  | Hs.77961 | Major histocompatibility complex, class I, B(*HLA-B*) |
|  | Hs.370937 | TAP binding protein (tapasin, *TAPBP*) |
|  | Hs.352018 | Transporter 1, ATP-binding cassette, sub-family B (MDR/TAP) *TAP1* |
| Antigen | Hs.278694 | CD209 antigen (*CD209*) |
| processing | Hs.77961 | Major histocompatibility complex, class I, B(*HLA-B*) |
|  | Hs.370937 | TAP binding protein (tapasin, *TAPBP*) |
| Antigen | Hs.278694 | CD209 antigen (*CD209*) |
| presentation | Hs.77961 | Major histocompatibility complex, class I, B(*HLA-B*) |
| Regulation of | Hs.535257 | Centaurin, beta 5 (*CENTB5*) |
| hydrolase activity | Hs.388034 | Retinoid X receptor, beta (*RXRB*) |
|  | Hs.470943 | Signal transducer and activator of transcription 1 (*STAT1*) |
| JAK-STAT | Hs.529400 | Interferon (alpha, beta and omega) receptor 1*(IFNAR1*) |
| cascade | Hs.470943 | Signal transducer and activator of transcription 1 (*STAT1*) |
|  | Hs.527973 | Suppressor of cytokine signaling 3 (*SOCS3*) |
