# Supplementary material for: Transcriptome profiling of the small intestinal epithelium in germfree versus conventional piglets
Source: BMC Genomics. 2007 Jul 5;8:215. doi: 10.1186/1471-2164-8-215 (PMC1949829; doi:10.1186/1471-2164-8-215)
Supplement: Additional file 6 — Marker genes identified by class prediction analysis. Table lists marker genes that distinguish germfree versus conventional ileal epithelia identified by class prediction analysis with GeneSpring software (Agilent). [file 1471-2164-8-215-S6.doc]

**Table S4: Marker genes identified by c**lass prediction analysis

| Category | Unigene ID | Gene description | Prediction strength^1^ |
| --- | --- | --- | --- |
| Differentially | [Hs.77961](http://www.ncbi.nlm.nih.gov/UniGene/clust.cgi?ORG=Hs&CID=77961) | Major histocompatibility complex, class I, B (*HLA-B*) | 13.34 |
| expressed | Hs.518424 | NADH dehydrogenase 1 beta subcomplex, 5 (*NDUFB5*) | 13.34 |
|  | Hs.524530 | CTD (carboxy-terminal domain, RNA polymerase II) small phosphatase 2 (*CTDSP2*) | 12.59 |
|  | Hs.133421 | Leukemia inhibitory factor receptor (*LIFR)* | 12.59 |
|  | Hs.352018 | Transporter 1, ATP-binding cassette, sub-family B (*TAP1*) | 11.73 |
|  | Hs.558922 | Mitochondrial coiled-coil domain 1 (*MCCD1*) | 11.73 |
|  | Hs.381099 | L-plastin ( *LCP1*) | 10.86 |
|  | Hs.509545 | Pre-B-cell leukemia transcription factor 2 (*PBX2*) | 10.86 |
|  | Hs.470943 | Signal transducer and activator of transcription 1 (*STAT1*) | 10.86 |
|  | Hs.449585 | Immunoglobulin lambda locus (*IGL@*) | 10.29 |
|  | Hs.490504 | Zinc finger protein 398 (*ZNF398*) | 10.29 |
|  | [Hs.534255](http://www.ncbi.nlm.nih.gov/UniGene/clust.cgi?ORG=Hs&CID=534255) | Beta-2-microglobulin (*B2M*) | 10.29 |
|  | Hs.389724 | Interferon-induced protein 44-like (*IFI44L*) | 10.29 |
| Not | Hs.507584 | RNA polymerase (*POLR1D*) | 11.73 |
| differentially | Hs.510409 | Cyclin K (*CCNK)* | 10.86 |
| expressed | Hs.447084 | Regenerating islet-derived 3 gamma (*REG3G*) | 10.86 |
|  | Hs.12646 | Poly (ADP) polymerase family member 12 (*PARP12*) | 10.86 |
|  | Hs.584884 | ATPase Ca^++^ transporting, type 2C member 1 (*ATP2C1*) | 10.62 |
|  | Hs.435741 | IQ motif and WD repeats 1 (*IQWD1*) | 10.29 |
|  | Hs.440414 | Spastic parapelegia 20 (*SPG20*) | 10.29 |

^1^Prediction strength measures the strength of association between class and expression level of the gene which is determined by hypergeometric probability (GeneSpring GX 7.3.1, Agilent Technologies).
