# Supplementary material for: Transcriptome profiling of the small intestinal epithelium in germfree versus conventional piglets
Source: BMC Genomics. 2007 Jul 5;8:215. doi: 10.1186/1471-2164-8-215 (PMC1949829; doi:10.1186/1471-2164-8-215)
Supplement: Additional file 7 — Sequences for qRT-PCR primers. Table lists the porcine-gene-specific primers used for qRT-PCR analysis. [file 1471-2164-8-215-S7.doc]

**Table S5: Sequences for qRT-**PCR primers

| Gene  Symbol | Accession  No. | Sense  primer 5’ to 3’ | Anti-sense  primer 5’ to 3’ | Amplicon  size |
| --- | --- | --- | --- | --- |
| *TLR4* | [AB188301](http://www.ncbi.nlm.nih.gov/entrez/viewer.fcgi?db=nucleotide&val=73760381) | CAAGGACCAGAAGCAGCTCC | GACGGCCTCGCTTATCTGAC | 121 (1901-2021)^1^ |
| *TLR9* | [NM_213958](http://www.ncbi.nlm.nih.gov/entrez/viewer.fcgi?db=nucleotide&val=47522745) | GGACCTCAGCTACAACAGCC | CGGCTATGGATGTCATTGTG | 120 (1647-1766) |
| *MYD88* | [AY435217](http://www.ncbi.nlm.nih.gov/entrez/viewer.fcgi?db=nucleotide&val=38565502) | CAGAAGCGACTGATCCCTGT | AGGATGCTGGGGAACTCTTT | 59 (29-87) |
| *TBK1* | [AJ952315](http://www.ncbi.nlm.nih.gov/entrez/viewer.fcgi?db=nucleotide&val=74369131) | TGAAATGGAGAAACAAGAGACCAC | GCCTTCGCAATGTTGACTGTC | 121 (637-757) |
| *IKBKE* | [BI342138](http://www.ncbi.nlm.nih.gov/entrez/viewer.fcgi?db=nucleotide&val=15035427) | GGCCTCACCACCCCTTATG | GCGGTTGTTGTCTTGGAGGT | 121 (294-414) |
| *NFκBIA* | [BI399403](http://www.ncbi.nlm.nih.gov/entrez/viewer.fcgi?db=nucleotide&val=15178464) | CCACACTTCAACAAGAGCGA | TCTTTGGGTGCTGATGTCAA | 110 (312-421) |
| *TOLLIP* | [CV870201](http://www.ncbi.nlm.nih.gov/entrez/viewer.fcgi?db=nucleotide&val=84124161) | GCTACGCCGTGTACGAGAC | GTAGAAGGAGTCCACGCCAG | 104 (381-484) |
| *NFκB1* | [BI347029](http://www.ncbi.nlm.nih.gov/entrez/viewer.fcgi?db=nucleotide&val=15040318) | TCGCTGCCAAAGAAGGACAT | AGCGTTCAGACCTTCACCGT | 101 (111-211) |
| *IRF3* | [NM_213770](http://www.ncbi.nlm.nih.gov/entrez/viewer.fcgi?db=nucleotide&val=47523299) | ACAAGCCTGACGGTGAGGTC | CAGAGGGTGTAGCGTGGTGAG | 121 (908-1028) |
| *IRF7* | [BP143360](http://www.ncbi.nlm.nih.gov/entrez/viewer.fcgi?db=nucleotide&val=40392831) | AAGACTCACCTGGGCAACGT | GGTGAGAGGAGAGCTGCGC | 126 (123-248) |
| *IRF9* | [CV868967](http://www.ncbi.nlm.nih.gov/entrez/viewer.fcgi?db=nucleotide&val=84122927) | ACCTCCTGCTCCCCTCTCTG | CCACCAATCAGGGTCCATG | 121 (271-391) |
| *IFNAR1* | [NM_213772](http://www.ncbi.nlm.nih.gov/entrez/viewer.fcgi?db=nucleotide&val=47523295) | AGCACATCTCTTTGGTCCGAA | GAGCACCGATACCGACACG | 121 (940-1060) |
| *IFNGR1* | [BX919556](http://www.ncbi.nlm.nih.gov/entrez/viewer.fcgi?db=nucleotide&val=90236625) | GTGTCAAGAGCCTCTTTGGCA | GAGGCTTTCACCGTTGCTGT | 121 (353-473) |
| *IFNGR2* | [CJ020667](http://www.ncbi.nlm.nih.gov/entrez/viewer.fcgi?db=nucleotide&val=54523973) | AAACAACAGCCGATGCGTCT | AGGCCTCTGAATTTCAGGACC | 121 (387-407) |
| *LIFR* | [U91518](http://www.ncbi.nlm.nih.gov/entrez/viewer.fcgi?db=nucleotide&val=2058661) | TCTCTCCAGTAGCTGAGCGTCC | GCTTCATCCACTCCTGGGTTC | 121 (371-491) |
| *PPARG* | [NM_214379](http://www.ncbi.nlm.nih.gov/entrez/viewer.fcgi?db=nucleotide&val=47523813) | GAATGTGAAGCCCATCGAGG | GCTTAGCAAAGAGCTGGGAGG | 101 (1284-1384) |
| *STAT1* | [NM_213769](http://www.ncbi.nlm.nih.gov/entrez/viewer.fcgi?db=nucleotide&val=47523305) | GTGCACGATGGTCTCAGCTTT | CCAGCAGTGGGACCAAGAAG | 121 (1475-1595) |
| *STAT2* | [NM_213889](http://www.ncbi.nlm.nih.gov/entrez/viewer.fcgi?db=nucleotide&val=47522707) | CCGGATCCTGGAATTAAGGG | AGAGTGTGTCTTCGCTGAGGC | 121 (441-561) |
| pan SLA I | [NM_213768](http://www.ncbi.nlm.nih.gov/entrez/viewer.fcgi?db=nucleotide&val=47523303) | GGAGGAGGAAACTGTGGCAG | TGTCTTTGGAGGATCTGACCG | 121(504-624) |
| *B2M* | [NM_213978](http://www.ncbi.nlm.nih.gov/entrez/viewer.fcgi?db=nucleotide&val=47522781) | TGTCTTTCAGCAAGGACTGGTC | GAGAGTCACGTGCTTCACGC | 101 (218-318) |
| *HSP70* | [M29506](http://www.ncbi.nlm.nih.gov/entrez/viewer.fcgi?db=nucleotide&val=164494) | GTATGAGGGTGAGAGGGCCAT | GGCATCAATGTCGAAGGTCA | 121 (550-670) |
| *SOCS3* | [AY785556](http://www.ncbi.nlm.nih.gov/entrez/viewer.fcgi?db=nucleotide&val=55535527) | GCCCCCCTAGAAGAGCCTATT | CCGTTGACTGTTTTCCGAC | 121 (464-584) |
| *IL10* | [NM_214041](http://www.ncbi.nlm.nih.gov/entrez/viewer.fcgi?db=nucleotide&val=47524185) | CTGGAAGACGTAATGCCGAAG | GCAGAAATTGATGACAGCGC | 121 (259-379) |

^1^Nucleotide position.
